# Working While Caregiving: A Scoping Review of the Experiences of Employed Female Caregivers

**DOI:** 10.1093/geroni/igaa057.2552

**Published:** 2020-12-16

**Authors:** Jessica King McLaughlin

**Affiliations:** University of Denver, Denver, Colorado, United States

## Abstract

The negative consequences of caregiving disproportionately impact women, as women make up the majority of informal caregivers and spend more time doing caregiving duties than men. The competing demands inherent in being a caregiver while working can increase levels of stress and burden that these individuals may experience. However, the nuances of the challenges of working female caregivers are still not salient. This review synthesizes the extant literature on working female caregivers to clarify the dilemmas they face and factors that impact their ordeals. Thirty studies on working female caregivers met inclusion criteria. Findings indicate common themes that emerged across the studies. These themes indicate the need to more comprehensively support the multifaceted nature of working female caregivers’ experiences in the workplace and outside of work. Furthermore, this review identifies areas in which informed policy can have a meaningful impact on working female caregivers’ lives.

